# Gendered forms of intergenerational solidarity in the COVID-19 pandemic: an intersectional perspective on the public renegotiation of a moral resource for future health crises in Germany

**DOI:** 10.1186/s12939-026-03014-7

**Published:** 2026-10-12

**Authors:** Eva Katharina Boser, Lena Dörmann, Niklas Ellerich-Groppe

**Affiliations:** Division of Ethics in Medicine, Department of Health Services Research, School for Medicine and Health Sciences, Carl von Ossietzky Universität Oldenburg, Oldenburg, Germany

**Keywords:** Intergenerational relations, Solidarity, Gender, Intersectionality, Ageism, Vulnerability, Moral resource, Health crisis, COVID-19 pandemic, Discourse analysis, Media coverage

## Abstract

**Background:**

During the COVID-19 pandemic, ‘intergenerational solidarity’ became a prominent buzzword in public debates. At the same time, a resurge of ageist stereotypes could be observed that proved to be a constant structuring element in the public renegotiation of intergenerational solidarity. Despite a growing body of research indicating that the COVID-19 pandemic also had a significant impact on inequalities related to other social categories, such as gender, the interplay between appeals for intergenerational solidarity and other social inequalities in public debates has received limited attention.

**Methods:**

To examine how appeals to intergenerational solidarity and gender-related aspects are intertwined in times of crisis, we provide an intersectional perspective on the public renegotiation of intergenerational solidarity during the COVID-19 pandemic, using the example of the German media debate. Based on a qualitative content analysis of articles from two major German weekly newspapers, *Die Zeit* and *Welt am Sonntag*, published between March 2020 and June 2022 (*n* = 351), we identified two paradigmatic cases for an intersectional in-depth-analysis, focusing primarily on the categorial intersection of age and gender within the material.

**Results:**

We can observe not only a resurge of ageism and age-related stereotypes in these media representations regarding various aspects of intergenerational solidarity, but also their entanglement with gender norms. Prevailing notions of intergenerational solidarity in the media continue to rely on gendered and age-based assumptions that shape how the moral actors, the basis, contributions, and normative goals of intergenerational solidarity are elucidated. We critically discuss these claims against the backdrop of broader intersectional feminist philosophy literature on structural social inequities and injustices.

**Conclusions:**

Our findings illustrate the significance of a gendered and heteronormative generational divide in appeals for intergenerational solidarity that include pertinent gender and age stereotypes. Intergenerational solidarity proves to be a moral resource in times of crisis, even though it often runs the risk of reiterating and perpetuating gendered ageism. However, approaches from intersectional feminist philosophy can help to conceptualize intergenerational solidarity as a diversity-sensitive moral resource in impending health crises in Germany and beyond.

**Supplementary Information:**

The online version contains supplementary material available at https://doi.org/10.1186/s12939-026-03014-7.

## Introduction: Intergenerational solidarity during the COVID-19 pandemic

During the COVID-19 pandemic, ‘intergenerational solidarity’ emerged as a prominent buzzword in public debates, often invoked as a means of fostering social cohesion and support across generations. Political and civil society actors, such as the AGE Platform Europe, pointed out the necessity to care for communities and to show solidarity across generations, stating: “COVID-19: with great challenge must come great solidarity” [1]. Similar appeals were made in global health contexts, for example, by the World Health Organization in discussions about vaccine development and distribution, which were influenced by a focus on the allegedly most vulnerable older generations [2].

The prominent role of intergenerational solidarity could, for example, be observed in Germany, where this concept was publicly invoked to justify political measures and the varying treatment of groups affected by the virus in different ways. Especially in the beginning of the COVID-19 pandemic, the protection of the supposedly vulnerable older population demanded restraint and sensitivity from younger people [3, 4]. As the COVID-19 pandemic unfolded, this rather unilateral idea of intergenerational solidarity as communal care was increasingly replaced by a conception of solidarity as mutual support between different age groups [3]. Nevertheless, debates on intergenerational solidarity became more and more controversial, with different underlying causes, shifting actors, addressees, contributions, and goals of solidarity being discussed. For example, significant differences could be observed regarding the starting point invoked, the assumed commitment, and the voluntary character of solidarity, underscoring the need for a thoroughly ethical analysis.

At the same time, the resurge of ageist stereotypes in many countries during the COVID-19 pandemic is striking (e.g., [5–7]). Thus, stereotypical representations of ageing for both younger and older people have proven to be a constant structuring element in the public renegotiation of intergenerational solidarity during the COVID-19 pandemic [8]. However, the research on growing ageism in the pandemic also emphasizes that ‘the young’ and ‘the old’ are heterogeneous groups and that, therefore, a sole focus on generations runs the risk of obscuring more salient social categories. Consequently, increasing attention has been given to the unequal impact of the COVID-19 pandemic on various social groups *within* generations. In particular, the COVID-19 pandemic exposed how deeply gender norms and social inequalities shape social attributions of vulnerability and responsibility. Early studies show that measures in pandemic combat had at least short-time effects on gender relations, for example regarding the gendered division of household labour and care work in many countries (e.g., [9–11]). Accordingly, warnings against a setback in gender equality due to the COVID-19 pandemic were issued, especially in the early stages of the crisis (e.g., [12]). In this context, marginalized communities — including LGBTQAI+ individuals — were found to face additional forms of exclusion and precarity [13, 14].

Despite this growing body of research indicating a major influence of the COVID-19 pandemic on inequalities related to other social categories, such as gender, the entanglement of appeals to intergenerational solidarity in public debates with further social inequalities has so far received only limited attention. However, a closer investigation of the connection between appeals to intergenerational solidarity and the role of gender within these calls is urgently needed to avoid gendered ageism and sustain solidarity as a moral resource in impending health crises.

Against this backdrop, we use the German media debate to explore how intergenerational solidarity was framed in public debates during the pandemic, particularly at the intersection of the social categories of gender and age. To this end, we offer an intersectional perspective on the public renegotiation of intergenerational solidarity in Germany during the COVID-19 pandemic to identify lessons for future health crises. After a brief clarification of the concepts of solidarity and generations, as well as a short overview of vulnerability, ageism and the gendered effects of the COVID-19 pandemic, we provide an intersectional analysis of two paradigmatic cases from the German media debate. Our findings illustrate the significance of a gendered and heteronormative generational divide in appeals for intergenerational solidarity that include pertinent gender and age stereotypes. In the subsequent discussion, referring to intersectional feminist philosophy literature on structural injustices and inequities, we outline perspectives on how to conceptualize intergenerational solidarity as a diversity-sensitive moral resource in impending health crises, such as the nursing crisis, to contribute to the development of sustainable healthcare in the future.

## Background: Intergenerational solidarity, ageism and gender effects in the COVID-19 pandemic

The COVID-19 pandemic can be understood as a stress test for the renegotiation of intergenerational relations in general, as well as for the potentials and pitfalls of intergenerational solidarity as a moral and political resource in health crises in particular [15]. The uncertainty surrounding the COVID-19 pandemic gave rise to controversial societal, political, and media debates in many countries about how intergenerational solidarity could contribute to pandemic control and societal cohesion in times of crises. In these debates, heterogeneous ideas were proposed regarding who should be in solidarity with whom and on what basis, conveying varying causes, motivations, levels of commitment and degrees of voluntariness for solidarity (cf., e.g., [3, 16–21]).

Despite the heterogeneity of interpretations of solidarity in public and academic debates, at least four constitutive elements of solidarity can be identified in these claims: solidarity refers to a situation in which a group or person, based on an identification grounded in a commonality, makes a contribution in or towards a group or person to pursue a normative goal (for this definition and for the following cf [22, 23]). The first element concerns the basis of solidarity: a person is not in solidarity with just anyone but rather establishes a specific relationship through *identification* based on a shared role, condition, lifestyle, or set of experiences [24–26]. The second element of solidarity is the *reference to a group*. One can distinguish between transitive and intransitive forms of solidarity: a person can be in solidarity within a group or towards a group or person ([27, 28] p. 62). However, solidarity comes at a cost; it always entails at least the willingness to make a *contribution*. This necessary contribution — through which solidarity is realized — has led scholars to emphasize the action-oriented character of solidarity [25, 26, 29] or to understand solidarity primarily as a practice (e.g., [30, 31]). Across these approaches, the actors of solidarity pursue a *normative goal* that guides their contributions and actions. Such a heuristic framework of solidarity allows to identify notions of solidarity in their whole plurality, to make them accessible for a distinct ethical analysis and an evaluation of their ethical legitimacy.

Following this heuristic framework, the fact that a member of a younger generation assumes a particular vulnerability of becoming severely ill with COVID-19 of their grandparents or other older persons in their immediate surroundings (*identification*) and tries to support (*contribution*) older fellow citizens (*group reference*) to sustain their health or wellbeing (*normative goal*) can be interpreted as an instance of solidarity from one generation toward another during the COVID-19 pandemic, conveying an understanding of solidarity as communal care. At the same time, recognition of shared suffering caused by the COVID-19 pandemic (*identification*) resulting in mutual support (*contribution*) among all generations (*group*) for the purpose of COVID-19 pandemic control (*normative goal*) represents another plausible interpretation of solidarity through the lens of this heuristic framework. Indeed, these different understandings of solidarity could be observed in public discourse during the COVID-19 pandemic in Germany [3]. They already point to another central concept that gained prominence in defining the moral actors and addressees of solidarity during the COVID-19 pandemic: the concept of ‘generation’, which likewise requires clarification.

With regard to how generations are conceptualized, three distinct perspectives can be identified [32–34], that imply different ideas of how intergenerationality, i.e., for the purpose of this paper, the moral relations between generations, can be spelled out [32]. From a *genealogical* perspective, generations refer to the succession of individuals and groups connected through descent. From a *chronological* perspective, they denote birth cohorts, that is, the stratification of people according to their year of birth (e.g., [35, 36]). Finally, from a *socio-cultural* perspective, generations are understood as collectives shaped by shared historical experiences, socio-cultural meanings, and processes of social (self-)ascription (e.g., [37, 38]). Generally, the generational perspective must be distinguished from the notion of age groups which delimit individuals “who fall within a certain age range or are at a certain stage of life” ([39] p. 12). At the same time, generations and age groups do overlap at a specific point in time. Accordingly, when discussing the chronological generations currently living, reference is often made to the older, middle or younger generations; in this view, age groups and generations coincide ([40] p. 45–46). This helps explain why public debates often conflate these perspectives, allowing ageist stereotypes to gain prominence in appeals for intergenerational solidarity [3, 8] and underlining the need to consider the category ‘age’ in investigations on generations.

Indeed, the resurge of ageist stereotypes during the COVID-19 pandemic has been a prominent topic in research as well [41–45], with the pandemic described as a “catalyst for ageism” [46]. In general, ageist practices or policies are based on the classification of people due to their chronological age and associated stereotypical images of ageing [47]. Such practices can result in stereotypical depictions, prejudices, or discrimination against different age groups. Ageism is often referred to in the context of older adults (e.g., [48]), however younger and middle-aged people can also face ageist treatment and stereotypical depictions [42]. It is possible to distinguish benevolent and hostile ageism: Benevolent ageism denies an age group’s competence and leads to patronizing behaviour, for example, in the form of the belittlement of older people and excessive offers of help. In contrast, hostile ageism disparages people through age-related prejudice and denies them their rights, ignores them, or mistreats them [48, 49].

In the reproduction of ageism during the COVID-19 pandemic (see, e.g., [5–7, 50]), both hostile and benevolent forms of ageism could be observed (e.g., [51, 52]). For example, ‘the old’ were frequently constructed as per se vulnerable risk groups, while ‘the young’ were portrayed as irresponsible infection spreaders ([4, 53] p. 1222, [54, 55]). These stereotypical representations of age groups were often linked to the attribution or neglect of vulnerabilities. For instance, in depicting older people as vulnerable and in need of support from younger generations, public debates during the pandemic tended to overlook the heterogeneity within the group of ‘the old’ [4]. At the same time, these debates ran the risk of obscuring potential vulnerabilities of younger generations who were also carrying heavy costs of pandemic measures, including closed schools and social isolation [56]. The pandemic thus highlighted the ubiquitous yet ambivalent intertwining of age(ing) and (attributed) vulnerability in public and academic discussions on health care [4]. Conceptually, one can distinguish between a general, inherent vulnerability as a human condition and a context specific-situational vulnerability ([57] p. 7–10). Inherent vulnerability refers to an ontological attribute of human life and the corresponding risks such as a disease or one’s own inevitable death; situational vulnerability focuses on the personal, social, political, or environmental factors and situations that make people (particularly) vulnerable [57]. Focusing on the sources and the production of vulnerabilities allows not only the question of who is vulnerable, but also of who is being made vulnerable due to actions, threats, harmful relations, and power structures. From this perspective, older people are not per se inherently vulnerable, but are made a vulnerable group through stereotypical, generalized classifications [4]. This understanding challenges age-related stereotypes in public debates and underlines the importance of analysing how age intersects with other social categories, thereby potentially generating distinct inequalities and vulnerabilities.

The need for such an integrated view on different social categories is also suggested by the public debates during the pandemic. Thus, beyond the central role of ageing and ageist stereotypes in the public discourse [45, 50, 58], the COVID-19 pandemic-induced inequalities were also evident regarding other social categories. Although the systematic discussion of the COVID-19 pandemic’s consequences is only beginning, first research indicates that — alongside e.g., *race* and *class* — gender is a particularly relevant factor shaping the burdens of the pandemic. Drawing on Butler’s understanding of gender as performative [59] and Young’s notion of gender as seriality [60], gender can be conceptualized as a social category that can be (self-)ascribed within a social context. This perspective emphasizes the socially constructed societal expectations arising from gender assignments and corresponding social power inequalities. Following such an understanding, far-reaching unequally distributed burdens of the pandemic can be identified, with Germany offering a particularly salient case in point. Thus, in line with findings from other countries, which often point to a slight increase or at least the persistence of gender differences in childcare (e.g., [61, 62]), informal care, and housework and caregiving more broadly (e.g., [10, 63–65]), as well as a tendency toward conformity with traditional gender roles (e.g., [66]), the gender effects of the pandemic on the distribution of care work in Germany are striking.

For example, Jessen and colleagues [11] observe an increase in the gender inequality in unpaid care work during the early stages of the COVID-19 pandemic. At the same time, this distribution gradually returned to pre-pandemic levels and a slight increase of couples sharing housework equally can be observed as the pandemic progressed. Although the authors classify these developments as temporary phenomena, the “moderate increase in traditionalization” in the beginning of the COVID-19 pandemic ([11] p. 657) is noteworthy, as it indicates the salience of gender aspects particularly in acute phases of crisis. These findings are consistent with the results of Bernhardt and colleagues [67] who examined the effect of working from home on parenting in the early stages of the COVID-19 pandemic and identified “strongly gendered patterns of changes in parenting” ([67] p. 935), thereby underscoring the need to consider gender when analysing the effects of the pandemic. In addition, Zwar and colleagues [68] found evidence of heightened attributed vulnerability among feminine informal caregivers during the COVID-19 pandemic, including higher levels of anxiety and lower quality of life, and thus a continuation of pre-pandemic patterns marked by a persistent gender gap in informal care. These findings are supported by a study by Klaus and Ehrlich [69] who report a significant increase in the proportion of women providing informal care between 2017 and the onset of the COVID-19 pandemic in 2020. In addition, pandemic measures in Germany have also been insufficiently able to address the fact that women often work shorter hours and are more likely to be in marginal employment arrangements, leading to a reinforcement of existing gender inequalities [70].

On the one hand, these findings must be situated within the specific context of the German care system, where home-based care provided by relatives – mostly perceived as ‘women’ – continues to be both the personally preferred and politically promoted care setting. In Germany, 84% of all those in need of care are cared for at home, and more than half of these are primarily looked after by family members [71]. At the same time, the discussion in Germany is also shaped by the specific organisation of the social security system as a system of social insurances, which are considered as institutionalised solidarity [22, 72]. Solidarity and subsidiarity have characterised the German welfare state system as normative principles since its inception, and they still influence debates about (health) care in Germany significantly [73, 74]. On the other hand, as seen above, the developments in Germany during the pandemic mirror those reported in other European countries, suggesting that, despite national differences, they reflect a broader trend in the gendered and generational distribution of care work. While age- and gender-based constructions of generations have thus proven to be decisive factors in COVID-19 pandemic-related inequalities, their entanglement and the specific inequalities resulting from it have not received much attention in research so far.

## Methods: An intersectional perspective on paradigmatic cases

To investigate the entanglement of appeals to intergenerational solidarity and gender in times of crisis, we adopt an intersectional lens. Intersectionality as an analytical framework was originally developed by feminist scholars in the 1980s in the United States. bell hooks made explicit that feminist claims and theorisations often solely represented the experiences of white women, without acknowledging the specific experiences of discrimination faced by women of colour [75]. In her research, the legal scholar and civil rights advocate Kimberlée Crenshaw highlighted the specific experiences of oppression faced by women of colour at the intersection of sexism and racism, and, therefore, at the intersection of the social categories of sex and race [76, 77]. Instead of adding or juxtaposing related experiences of systematic oppression due to different social categories, Crenshaw used the metaphor of an intersection to understand specific experiences of discrimination linked to different, converging social categories (ibid.). Such an intersectional perspective enables a deeper understanding of different social positions/identities linked to social inequalities and can thus reveal different forms of individual, institutional, or structural discrimination [78, 79], as long as – and only if – the inherent variety of experiences in group formation processes is considered [80, 81]. Today, intersectionality serves as an analytical framework across disciplines for examining how various social categories, such as sex, gender, disability, age, and class, intersect within broader systems of power, thereby influencing individuals’ exposure to sexism, racism, discrimination, and privilege [77, 80].

In our analysis, we understand intersectionality as a work in progress rather than a fixed theoretical framework, with the aim of “mov[ing] intersectionality to unexplored places” ([82] p. 305). While intersectional perspectives have already been used to uncover gendered racism in the COVID-19 pandemic [83], the present study applies an intersectional analysis to the specific intersection of gender and age in the context of media debates on intergenerational solidarity during the COVID-19 pandemic. Given the bioethical significance of media debates [84], media contributions provide a fruitful starting point for investigating public renegotiations of intergenerational relations, as well as social relations in general in times of crises, as the growing corpus of media debate analyses on the COVID-19 pandemic-related topics illustrates (e.g., [16, 85, 86]). In our analysis, we focus on Germany as a particularly salient case in point. Thus, referring to ‘solidarity’ has a long tradition in debates on social and health policy in Germany. Indeed, the concept has proven to serve as an anchor term in discourses during the COVID-19 pandemic as well (see e.g., [8, 16, 23]). Furthermore, the German media landscape fosters the public (re)negotiation of bioethical questions in general and can also be understood as a factor that shapes bioethical debates in Germany ([87], pp. 986–987). Hence, we can also expect a particularly multifaceted discussion of the entanglement of intergenerational solidarity and gender aspects in the German media discourse. At the same time, despite the specific circumstances in Germany, comparable trends to those in other countries could be observed regarding the categories of age/generation and gender, making the German debate a promising case for an initial exploration of the discursive mechanisms of intersectional discrimination in the context of intergenerational solidarity.

Our analysis is based on a corpus drawn from two nationally influential weekly newspapers. *DIE ZEIT* and *Welt am Sonntag* are widely regarded as leading media outlets that observe, interpret, and contextualize social developments, thus offering a productive source for examining evolving public debates [88]. Both newspapers reach large, heterogeneous readerships and combine in-depth commentary with broader journalistic coverage, making them well suited to trace public negotiations of intergenerational relations during the COVID-19 pandemic. For this study, we systematically collected articles published in print and online editions of *DIE ZEIT* and *Welt am Sonntag* between March 2020 and June 2022. This period encompasses several phases of the COVID-19 pandemic in Germany and therefore captures shifting dynamics of public discussions under conditions of prolonged crisis [89]. Articles were retrieved from the publishers’ digital archives. To identify relevant material, we applied the following search string:

((Covid OR Corona OR Pandemic) AND (solidar * OR cohesion* OR connectedness* OR justice* OR “personal responsibility” OR “social responsibility” OR conflict) AND (generation* OR cohort* OR age group OR working* OR “old and young” OR “the elderly” OR “the young” OR “the old” OR “older people” OR “young people” OR “young adults” OR senior* OR pension*/retire* OR older* OR nursing home OR school* OR student* OR younger* OR youth* OR child*))^1^

In addition, we applied specific inclusion and exclusion criteria, such as the exclusion of short notices, articles shorter than 300 words, pieces with low thematic relevance, and letters to the editor. After removing duplicates and assessing thematic plausibility and relevance, the final sample consisted of 351 articles.

The material was analysed following the principles of qualitative content analysis [90]. Categories were derived using the software MAXQDA24 through a combination of deductive and inductive approaches. To ensure internal quality in the coding process, we integrated the principle of intercoder agreement within the research team at different stages of the analysis. After completing the second round of coding, we applied an in-depth case-to-case analysis to identify paradigmatic cases [90]. These cases represent recurring patterns or phenomena within the corpus and thereby illustrate broader analytical dimensions of a research topic [91]. To identify these paradigmatic cases, we analysed the multidimensional relationships between coded segments across the categories *gender*, *solidarity*, and *generations*, as well as their respective subcodes. We used cross-tabulations to synoptical compare the named categories within each coded segment of the 351 articles in our major sample. Emerging cases were subsequently compared to identify commonalities and differences, and to determine paradigmatic cases that exemplified a recognised phenomenon in relation to the research question.

For this study, we identified two paradigmatic cases on caring relations, which represent forms of intergenerational solidarity that emerged prominently from both the empirical material and the broader analytical process described above. These cases provide a prototypical perspective on the intersection of gender and age. We used these two cases to compare pertinent characteristics of intergenerational solidarity. Here, the previously outlined elements of solidarity (*identification*,* group*,* contribution*,* normative goal*) served as anchor points for the analysis. In addition, the three conceptualizations of generations (*genealogical*,* chronological*,* and socio-cultural*) provided a framework for contrasting references to generations within the material. However, when categorizing these generational differences, we had to consider the plurality within generational realities to adequately grasp potential and existing inequalities.

## Results: Gendered forms of intergenerational solidarity

In our analysis, we identified two paradigmatic cases of caring relations during the COVID-19 pandemic in Germany. By applying an intersectional lens, we were able to delineate the gendered ageist dimensions of these forms of intergenerational solidarity. The first paradigmatic case, published in *DIE ZEIT* on July 9, 2020, under the title “Take care!”, written by Christiane Grefe (in the following quoted as *Case 1*), focuses on institutionalized eldercare in nursing homes as well as care arrangements within families in general. The article portrays eldercare as a system in crisis, with the COVID-19 pandemic exposing and exacerbating long-standing structural deficits, such as staff shortages and excessive bureaucratic regulation. In several passages, the author adopts a personal perspective, sharing first-hand experiences of caring for her mother within a failing care system. The article also highlights a general lack of intergenerational awareness and engagement with the issue of eldercare. Moreover, the author critically reflects on the foundations of the German care system, which remains implicitly based on traditional family and gender roles, particularly the assumption of women as primary caregivers.

The second paradigmatic case, published in *Welt am Sonntag* on December 20, 2020, and titled “Let us talk”, written by Jennifer Wilton and Eva Marie Kogel (in the following quoted as *Case 2*), approaches the topic of care through an explicitly intergenerational lens. Presented as an interview with four individuals from different age groups and social backgrounds, the article addresses caregiving across various contexts, including family life and the workplace. It illuminates the diverse ways in which care responsibilities are distributed and experienced, placing particular emphasis on the persistent gendered burden of care. Beyond gender, the article also highlights intragenerational inequalities, noting that the crisis disproportionately affected those in precarious employment, especially among younger and middle-aged generations.

Applying an intersectional perspective on gender and age, in combination with the heuristic framework of solidarity and its four analytical elements (*identification*,* group*,* contribution*, and *normative goal*), allows for a reconstruction of how different understandings of solidarity are articulated in the media renegotiation of intergenerational solidarity and how the categories of age and gender shape these representations. In *Case 1*, intergenerational solidarity is spelled out as follows: familial relations (identification) should motivate younger and middle-aged relatives (group 1) to engage in care actions (contribution) directed toward weaker (i.e., older) generations (group 2) to promote their well-being (normative goal). In *Case 2*, we can broadly identify intergenerational relations in familial and broader societal contexts (e.g., workplace) (identification) that form the basis for care practices (contribution) carried out primarily by middle-aged women (group 1) for children and older relatives, as well as colleagues (group 2) to support them in their needs and well-being (normative goal). These findings are presented in greater detail below.

### Identification: We are family – but are we in solidarity?

Regarding the first element of *identification*, both paradigmatic cases reveal a strong focus on family ties — thus reflecting a predominantly genealogical understanding of generations — that serve as the starting point for intergenerational solidarity. This becomes particularly evident in the first paradigmatic case from *DIE ZEIT*, where solidarity is framed primarily through close family relations, especially in mother-daughter ties. Thus, the author describes how, to care for her aging mother, she commuted long distances for years — motivated by familial duty and affection, but also due to the insufficient support of the care system in Germany. She states: “The wife or a daughter usually provides care alone, with 24/7 availability”^2^ (Case 1, p. 33). With this statement, the author indicates kinship relations as a starting point for intergenerational solidarity and care, on the one hand. On the other hand, it conveys the impression that this basis of identification appears to depend on the feminine members of the different generations, underlining the continued salience of gender aspects in the motivation for intergenerational care.

The second paradigmatic case from the *Welt am Sonntag* extends this perspective by placing greater emphasis on the broader challenge of maintaining social embeddedness across generations within kinship ties as well as the work context, or structures of social equality in general. While kinship ties remain an important point of reference, the article draws attention to the societal structures as a whole that implicitly presuppose and rely on these intra-familial care relations. Unlike the first case, which focuses mainly on care for older generations, care for the younger generations, i.e., the own children, moves to the foreground. The pandemic proves to be a reinforcing factor here, where the traditional gendered division of care becomes particularly visible, as the following statement from an interviewee illustrates: “I have to say that I actually enjoyed the lockdown in a way, at least the first time around. I had more time with my children. Especially when you work, there are many things in everyday life that get neglected. Of course, it was more difficult at work […]” (Case 2, p. 15). The pandemic constellation is thus perceived simultaneously as an opportunity for intensified care interactions and as a challenge due to precarious or destabilized working conditions, that are both shaped and influenced by age- and gender-related experiences. It becomes obvious that this concurrence of opportunity and challenge has to be handled by women — or, more specifically, by mothers — rather than by all parents in the same way. From an intersectional perspective, the burden on young parents — which was regularly highlighted in public discourse — thus appears to be a rather one-sided reliance on women’s care work. At the same time, the socio-economic status emerges as another social category, here, that plays a significant role in the social positioning of those acting in solidarity. Both cases thus reflect an ambivalent recourse to gender and age aspects: While gendered ideas of the succession of kinship generations prove to be solid and obvious bases for identification and group adherence, the risk of a reinforcement of traditional gender- and age-related roles stands out.

The effectiveness of gender and age stereotypes regarding the basis of solidarity becomes even clearer if one focuses on how the different age groups are portrayed in the paradigmatic cases. For example, the author of the first case points out, that younger people distance themselves from older people, as they do not identify with their care needs. In the material, this distancing is linked to a general tendency to avoid engagement with ageing as a life phase, despite a growing openness towards discussions of death and dying (Case 1, p. 33). This reveals a clear distinction: While ‘the young’ are presented as capable and healthy group, old age is linked to frailty, death and dying. The middle-aged — one could conclude — are in between in every respect. At the same time, it is striking, that the author uses terms such as “daughters” to further substantiate the younger generations (e.g., Case 1, p. 33). As a result, the subtle criticism of the younger generation becomes an accusation aimed specifically at the feminine members of that generation — the ageist stereotype manifests as gendered.

Finally, the effectiveness of age stereotypes in general becomes evident in the criticism of a lacking overarching point of identification for solidarity, that is articulated in case 2. Here, the authors advocate for a renewed dialogue between generational groups, aiming to reconcile their differing needs in the context of the COVID-19 pandemic rather than framing generations in opposition. Thus, one interviewee states: “My hope is that we will come together again. That this division in Germany and around the world, which has been getting worse and worse, will perhaps diminish again. I hope that this crisis will make us realize that things cannot continue as they are at the moment. Instead, we must come together” (Case 2, p. 15). Besides the fact, that the pandemic is understood as a crucial potential turning point in time for intergenerational relations, this passage underscores the interdependence of generations, both within families and across society as a whole — and understands them therefore as mutually obligated to solidarity. At the same time, it becomes apparent that currently solidarity takes its starting point rather in age- and gender-stereotypical depictions of generations and family ties, that serve as a productive, yet ambivalent starting point for intergenerational solidarity in the COVID-19 pandemic. However, that the allegedly general appeal to all generations is in fact gendered, becomes apparent when we turn to the elements of the group and the contribution in the notions of intergenerational solidarity.

### Group: The middle-aged women as solidary subjects

Regarding the element of the *group*, both paradigmatic cases convey a rather transitive idea of intergenerational solidarity, in which one generation acts in solidarity towards another generation. Here, in line with the point of identification, the significance of family contexts and genealogical ties becomes evident. At the same time, the actors and objects of solidarity are also addressed in chronological and age-group related terms — as ‘the middle-aged’ or ‘the young’.

In the first paradigmatic case from *DIE ZEIT*, ‘younger’ generations are described as withdrawing from collective responsibility, in the sense of not standing up for the public cause of good eldercare, as the following excerpt outlines: “But there is no such thing as ‘Thursdays for Care’. The reason: the old themselves can no longer walk. Relatives and caregivers don’t have time. And when no one marches ahead, daughters, grandchildren, and neighbours do not join in either” (Case 1, p. 33). First, by referring to ‘daughters’, the author implicitly frames caregiving as a ‘feminine’ task, once again emphasizing the effectiveness of gender aspects in this debate, passed down from one generation to the next and expected across different age groups, based on the gendered stereotypical perception of ‘the caring daughters’. Second, the quotation emphatically highlights middle-aged individuals, particularly women, as primary subjects of solidarity. Third, it implies a criticism of ‘younger’ generations, which, according to the author, do not adequately perceive themselves as part of the intergenerational community. However, as the ‘younger’ generations are prominently addressed as ‘daughters’, young women are obviously more likely to be expected to take on caregiving responsibilities. Finally, ‘the old’ become apparent as addressees of intergenerational support and are depicted in terms of frailty, disability, and decay, as they “can no longer walk” (Case 1, p. 33). From an intersectional perspective, this paints a picture of a transitive solidarity, where the ‘feminine’ members of the younger generations, i.e. daughters and mothers, are understood as the subjects of solidarity that care for the — stereotypically presented — older generations and, in this way, sustain family life during crises.

In the second paradigmatic case, an interviewee explicitly points out this one sided, gender-related burden: “[W]omen were and still are way more stressed out. So much for equality” (Case 2, p. 7) and continues: “It would be good if the government could see what moms are doing and support them more, especially single parents” (Case 2, p. 15). Caregiving within genealogical ties is thus consistently framed as a gendered responsibility, stereotypically connoted as ‘feminine’, with middle-aged women expected to ensure continuity of care for children and grandparents despite limited institutional support and significant personal cost. On the one hand, these quotations testify to unequal *inter*generational burdens, as the middle-aged generation appears to carry a disproportionate share of care responsibilities during the COVID-19 pandemic. On the other hand, it even points to unequal *intra*generational burdens, demonstrating that the middle-aged solidary subject is — in the most cases — feminine. Hence, particularly middle-aged women as a socially constructed generational group are depicted as solidary and responsible actors towards other age groups, while being potentially overburdened and vulnerable objects of gendered ageism. Here, gendered ageism serves as a stereotypical portrayal in public media discourse, normalising the expected self-sacrificing attitude of the social group of middle-aged women. An intersectional lens focusing on gender and age thus reveals the effectiveness and reinforcement of patriarchal power structures in the public discourse that can stigmatise different age groups through gendered ageism.

### Contribution: “Women have always been systemically relevant” – The ‘feminine’ care of solidarity

In terms of *contribution*, our case from *DIE ZEIT* foregrounds care provided to older relatives and the support extended to overworked healthcare professionals, portraying women as simultaneously responsible for both familial and societal care and thus embodying a double role. The author explicitly criticizes the outdated structure of the care system in Germany that relies heavily on family members. However, from the overall context of this statement, it becomes evident that she especially thinks of the feminine relatives here. Thus, due to the staff shortages in nursing homes, the author — as a daughter — steps in to care for her mother while also compensating for the lack of resources among nursing staff, as the following quotation illustrates: “So while I took another spontaneous vacation and fed her spoonful by spoonful, meal by meal, I noticed that after changing her diapers, the nurses sometimes forgot to disinfect their hands. Understandable — the beeper was beeping” (Case 1, p. 33). This passage not only conveys the concrete nature of expected care contributions in practice but also highlights their character as compensation for systemic failures that are offset by the (unpaid) work of – once again – middle-aged women. Besides the systemic failure, the author makes clear that she sees another reason for the one-sided burden on this age group in a younger generation that distances itself from older people and does not identify with their care needs (Case 1, p. 33). The lack of identification of the younger generation has thus concrete consequences regarding the expected contributions: Because the other generations do not feel responsible, the burden falls on the middle-aged.

The second paradigmatic case from *Welt am Sonntag* extends this depiction, again emphasizing the double burden borne by middle-aged women. One of the interviewees describes her responsibility for caring for her children while also supporting employees of her business, which she was forced to close again during the second lockdown. While acknowledging the necessity of lockdown measures to protect society as a whole, she states: “I just think: this is how it is now, we have to make the best of it. You cannot change the facts. Complaining is not my thing” (Case 2, p. 15). This statement underscores the (double) burden placed on middle-aged women that is nevertheless perceived as an unavoidable, nearly ‘natural’ contribution, thereby underlining the seemingly inevitable gender stereotypical division of care work linked to this specific stage of life. As a result, the allegedly ‘natural’ intergenerational care remains either politically invisible in its gendered form or is criticised as largely absent at all.

At the same time, the second paradigmatic case highlights the gendered character of these stereotypical representations of ageing also beyond the pandemic. The article suggests a shift in gendered responsibility to care and provision onto women in times of crises. This becomes visible in the following statement by one of the interviewees: “In times of crisis, people always remember that there are women, and that they have to fix things. Are women suddenly systemically relevant now? Women have always been systemically relevant” (Case 2, p. 15). This remark can be understood as a specification of contribution expectations that are not assigned to all middle-aged persons, but primarily to middle-aged *women*. How deeply these expectations are embedded in public consciousness becomes apparent in a quotation that emphasizes the exceptionality of a male caregiver: “In principle, you, Sven, do what usually women do” (Case 2, p. 15).

Hence, contribution expectations formulated under the concept of generation ultimately prove to be, at least in part, an expression of interwoven age stereotypes and gendered role expectations. Younger persons are stereotypical portrayed as more distant from caregiving issues, older persons as dependent on support, and middle-aged women as primarily responsible for maintaining care relations between generations and carrying the major burden.

### Normative goal: Sustaining care relations and societal cohesion at all

Regarding the *normative goal*, in the first paradigmatic case from *DIE ZEIT*, the author frames solidarity as promoting well-being, health, dignity, and justice within a specific kinship setting, exemplified by a daughter caring for her older mother. The author links this orientation to structural conditions of the German care system that is described as inadequately equipped for an increasingly aging society in which “more families are moving apart, […] women are working and the state is withdrawing” (Case 1, p. 33). The normative orientation is thus presented as depending on women who are enabling ageing in dignity despite limited institutional support.

In the second paradigmatic case from *Welt am Sonntag*, the normative goal of solidarity extends beyond familial ties. This is achieved not only through the dialogue-based nature of the article, but also by encompassing a broader social contract across generations. This contract is aimed at ensuring equal opportunities and fostering trust at both the individual and structural levels. This is illustrated by an interviewee who provides care for his older parents-in-law through everyday assistance, such as shopping, while emphasising that care also involves social and emotional dimensions, noting that “[t]hey need personal contact” (Case 2, p. 15).

Across both articles, the normative goal of solidary actions is represented as intergenerational exchange and support of other persons/groups. At the same time, existing injustices within the broader societal structures can be reconstructed in the material, as the current care system is unable to provide adequate care for all. For example, in the first paradigmatic case, the author argues that, regardless of socio-economic resources for professional support or the capacities of relatives to step in, care and ageing should be a concern for all generations (Case 1, p. 33). Moving beyond the genealogical structured family care context, the second paradigmatic case emphasizes the importance of broader social cohesion in times of increasing individualization. It draws attention to groups whose needs are often overlooked (e.g., unemployed persons or artists without commissions), noting that such neglect reinforces the assumption that responsibility rests primarily with the state, though “it starts with the people, not ‘up there’” (Case 2, p. 15).

Although these normative goals may appear universally valid at first glance, an intersectional perspective reveals that both their definition and, in particular, their realization involve far-reaching age and gender aspects. For example, in Case 1, it is explicitly the responsibility of persons socially perceived as ‘women’ to compensate failing institutional support in eldercare. The quotations further demonstrate that the concrete purposes of (feminine) intergenerational care are bound to binary heterosexual gender and age roles and extend beyond caring for one’s own relatives. Rather, they take on a societal dimension in which feminine solidary care appears as a systemic necessity and as a form of social glue that holds society together in times of crises.

## Discussion: Gender, generations and solidarity in crises

The results of our intersectional analysis of two paradigmatic cases from the German media debate underline the significance of different social categories within appeals for intergenerational solidarity in times of crises. Thus, we can observe not only a resurge of ageism and age-related stereotypes in these German media representations, but particularly their entanglement with gender norms during the COVID-19 pandemic.

While the first paradigmatic case from *DIE ZEIT* focuses primarily on solidarity within the family (Case 1), the second from *Welt am Sonntag* emphasizes broader social ties (Case 2). Across both cases, people perceived as women — especially ‘middle-aged women’ — are depicted as central caregivers who carry a double burden of private and societal care while younger generations seem more detached from care-related concerns. During the lockdowns, the retreat into the private sphere obscured once again intergenerational social responsibility and the duty to act for the common good. At the same time, persistent stereotypes of ageing become apparent in the public renegotiation of intergenerational solidarity in our media analysis, for example in depictions of older people as weak, dependent, or solely as objects of care and as being in a phase of life perceived as less worth living. Normative goals range from promoting dignity and justice to strengthening a wider social contract across generations. Overall, the material underlines that prevailing notions of intergenerational solidarity in the German media coverage seem to rely on gendered and age-based assumptions that shape how vulnerabilities, responsibilities, and moral claims are assigned.

These findings point to two important aspects that deserve further discussion: (1) the gendered character of the ageist stereotypes during the pandemic, and (2) the prevalence of gendered ageism as structuring element of notions of intergenerational solidarity:

1. It becomes apparent, that the discursive reference to generations is permeated with age stereotypes. One pertinent example is the general attribution of vulnerability to older age groups in both cases examined. This empirical finding is in line with other studies on the resurge of ageist stereotypes in (media) debates in Germany and beyond during the pandemic [8, 43, 45, 54]. At the same time, this finding is consistent with the research on age stereotypes in general, according to which ageing is commonly perceived negatively from an early age [47, 92], potentially leading to intergenerational conflict [53]. On a conceptual level, our results underline a neglected consideration of the heterogeneity of age groups in public discourses. Thus, as Langmann suggests, we could observe in our analysis how older people *were made* vulnerable as a collective due to their stereotypical representation in the media [4]. This is consistent with other diagnoses on ageism in the pandemic, suggesting that mainly older people faced hostile (and less benevolent) forms of ageism, for example, by being deprived of competences [93]. Thus, also in our material, it becomes evident that stereotypical representations of ageing play a crucial role in attributing vulnerability, responsibility, and capabilities to specific age groups.
  At the same time, our analysis makes clear that the resurge of ageism during the COVID-19 pandemic, and the characterization of the pandemic as a “catalyst for ageism” [46], can only be adequately understood through an intersectional perspective. Thus, this approach allowed us to carve out the gendered character of different forms of supposedly general ageism within the appeals for intergenerational solidarity that would otherwise remain invisible, such as the idea of a middle-aged daughter caring for their parents and children. These findings indicate the salience of the category ‘gender’ in the public renegotiation of intergenerational solidarity in times of crises, resonating the pertinent research on the unequal gender effects of the pandemic (cf., e.g., [11, 67–69]). As a result, notions of intergenerational solidarity in the German public discourse can be understood to operate within a binary gender framework and to perpetuate gendered attributions within caregiving practices and broader social developments. The at least temporary backlash to traditional role models and stereotypical gender representations in many countries and their discursive appeal underscore the effectiveness of these stereotypes and how deeply they are embedded in public consciousness. It is essential to consider this intertwining of gender and generational issues and the looming threat of a return to traditional roles in the urgently needed reform of the care system as a whole to avoid the perpetuation of social inequalities within heteronormative gender roles.
  At the same time, life realities that did not fit into the heteronormative image of nuclear-family care as well as temporalities of ageing independent of genealogical notions of generations could not be identified in the media representation and remained marginalized. This blind spot becomes especially apparent if one considers queer conceptions of time and care that challenge patriarchal and stereotypical embedded notions of gender and age bound to the heteronormative reproductive life cycle [94–97]. While based on these approaches mentorship, neighbourly assistance, and caregiving within friendships can be understood as valuable forms of intergenerational solidarity, they are not represented in the public discourse, once again highlighting the stereotypical character of the analysed media debate. This observation demonstrates that an intersectional perspective allows to carve out the effectiveness of fundamental structural power imbalances in the public discourse that shape stereotypical perceptions of gender and age as well as corresponding social expectations and responsibilities through social attributions in healthcare and beyond [49, 98]. In this way, it can contribute to estimate the so far often neglected influence of intersecting identities in ageism [49], avoid simplified understandings of social groups tied to specific practices, and promote health equity and justice [99].
2. Gendered ageist stereotypes proved to be a constant influencing factor across all elements of solidarity (i.e., identification, group, contribution, and normative goal) in our analysis. This poses the question to what extent the allegedly encompassing intergenerational solidarity can be understood as a normative valuable moral resource in times of crises – or whether the appeals to solidarity between the generations run the risk of having opposite effects on societal cohesion by reinforcing gendered ageism or causing new moral problems. At the same time, a critical assessment of the gendered age stereotypes embedded in the appeals to intergenerational solidarity through the lens of intersectional feminist philosophy literature on structural social inequities and injustices allows for the development of initial ideas toward a diversity-sensitive conceptualization of intergenerational solidarity as a moral resource in times of crises.
  With regard to *identification*, the material conveys the effectiveness of strongly ageist and gendered reference points and expectations, particularly the persistent ideals of the ’good mother’ and the ’good daughter’, that are linked to specific genealogical ties and age-related stereotypes, risking to conflict with personal self-understandings. From an intersectional feminist perspective on structural social inequities and injustices, three aspects are particularly relevant for broadening the basis of identification of solidarity and avoiding further moral problems: (a) challenging stereotypical and essentialist conceptions of gender, (b) accounting for additional axes of marginalization, and (c) conceiving generational identification beyond kinship relations.

- The prominence of gendered ageist stereotypes in the public media discourse points to underlying essentialist understandings of gender that presume shared characteristics as a precondition for rendering the category ‘women’ intelligible. This tendency is also evident in our material. Such assumptions can be challenged by drawing on feminist theorists such as Witt [100] or Stone [101], who offer more dynamic accounts of gender and social belonging. Adopting this perspective may help to avoid reinforcing existing gendered ageisms and to expand the basis for intergenerational solidarity. More broadly, these findings invite a more fluid understanding of gender (roles) and a critical reflection on the epistemic conditions shaping individuals’ positioning in relation to social groups [79]. From the intersectional perspective on our material, identifying with specific gender or age identities should not create a norm and narrow the basis for identification; rather it should serve as starting point of belonging and building solidarity across social differences.
- The consideration of further axes of marginalization can broaden the basis of *identification*. While the intersectional perspective on age and gender has already enriched our understanding of intergenerational solidarity in the discourse, the material simultaneously points to the limitations of a discourse in which other structural inequalities and social positions remain largely invisible. Building on Hill Collins [78] and Alcoff [102], our analysis could be extended by integrating additional intersectional categories, such as race and class, to better understand self-perceived and socially imposed identification incentives within solidary actions. This could enable a broader perspective on the complexity of marginalization, for example regarding the recognition of specific realities of life, knowledge production, and experiences of racialized communities (e.g., [102–104]).
- In our analysis, we witnessed a reinforced *identification* within kinship relations during the COVID-19 pandemic, with intergenerational solidarity predominantly framed through family relations and caregiving responsibilities. In doing so, the public discourse tended to privilege heteronormative and family-centered understandings of solidarity. An intersectional perspective highlights the need of fostering identification beyond kinship structures, encompassing broader social relationships and structures (e.g., friendships, neighbourhoods, broader society, across borders). This may help to address social inequalities and to consider intragenerational heterogeneity in a comprehensive way.

Taken together, these three aspects emphasize — in line with Chung [98] and Dodds [105] — the importance of considering shared human vulnerability as a basis for intergenerational solidarity in healthcare, without neglecting the situatedness and diversity of specific groups and experiences. In this regard, the COVID-19 pandemic and the critical assessment of the media discourse could serve as an example of how shared historical contexts and experiences can create common frames of reference for identifying with specific social groups and standing up for one another, without making generalisations about shared characteristics, such as what defines the group of women [101].

At the *group level*, the intersectional approach demonstrates that neglecting inter- and intra-group diversity risks reinforcing processes of age and gender stereotyping, potentially leading to othering. In particular, the media discourse tended to position some groups primarily through vulnerability and dependency while others appeared predominantly associated with caregiving-responsibility and social cohesion. In our material, especially the stereotypical depiction of ‘middle-aged women’ was present. However, to sustain intergenerational solidarity as a diversity-sensitive moral resource in times of crises, it is crucial to avoid reproducing a purely binary understanding of gender that excludes queer care relationships and solidarity within or towards different groups [59, 96]. Rather, it is necessary to differentiate and situate social standpoints to prevent epistemic and structural injustices within broader power structures [102, 106–108].

Yet an intersectional perspective also reveals the ambivalence and norms within group formation processes in relation to gender and age. While social groups are necessary for making structural inequalities visible, they simultaneously risk producing new forms of exclusion mechanisms ([109] p. 17). This means, while group formation processes should remain as inclusive as possible, in order to achieve a political goal, it might be justified to define group traits, without devaluing and creating vulnerable positions. In this sense, references to socially burdened or vulnerable groups may contribute to making unequal distributions of care responsibilities and structural disadvantages visible. Our analysis of the German media discourse during the COVID-19 pandemic illustrates this ambivalence particularly through the depiction of ’middle-aged women’ as a vulnerable, overburdened caregiving group. At the same time, the findings highlight the conceptual importance and the need for nuanced attributions of vulnerability in the context of assessing forms of intergenerational solidarity regarding social categories beyond age and gender in future health care crises scenarios.

In terms of *contribution*, the material demonstrates how gendered caregiving responsibilities are linked to entrenched social expectations, particularly during the COVID-19 pandemic. Thus, the solidarity of — allegedly all individuals of — the ‘young’ or ‘middle-aged’ generation with the ‘vulnerable’ turned out to be a one-sided burdening of only a part of this generation, i.e., all individuals perceived as ‘women’. This resonates the insight brought forward by intersectional feminist perspectives that in the binary scheme women bodies often occupy less privileged social positions compared to men, creating sexist injustices [110]. Through an intersectional lens, the effectiveness of gender stereotypes in the one-sided attribution of pandemic burdens to primarily middle-aged women in intergenerational solidarity becomes apparent, thus showing how overlooking the effectiveness of social categories leads to their reinforcement. Although such a perspective provides an important foundation for analysing structural sexism in relation to social positions, a sole binary perspective on gender risks to exclude other identities, e.g., trans* identities [111, 112]. This becomes also apparent in our material, where the diversity of care relations, e.g., those that extend beyond heteronormative family structures to include queer and community-based forms of care, remains insufficiently recognised and institutionalised in the media debate.

Besides the question of the legitimacy of an attribution of contributions, the capability and opportunity to contribute must be considered. An unreflected assignment of contributions always runs the risk of overburdening individuals. Therefore, it is necessary to take into account the structural conditions of social positions and affiliations ([108] p. 56–59]. For instance, Youngs conceptualization of structural injustice and responsibility [108, 113] can be used to argue for an appropriate allocation of social responsibilities that consistently considers the structural aspects of intergenerational relationships and, presumably, clear group boundaries. This can provide the basis for a fair distribution of burdens in healthcare [114]. Our critical discussion of the different gendered forms of intergenerational solidarity in the public debate thus highlights the need for a diversity-sensitive and anti-discriminatory distribution of caregiving responsibilities and burdens across society [97, 115].

Finally, the *normative goal* of intergenerational solidarity has to be assessed. The material from our media analysis pointed to the role of intergenerational solidarity as a normative basis for good (health) care relations and societal cohesion. While this normative goal may, at first glance, be justified, an intersectional perspective calls this into question. Thus, the normative goal remains incomplete as long as care work continues to reproduce dominant power hierarchies and patriarchal structures. Especially if one considers the way how the normative goal is achieved, it becomes evident that its achievement relies on unequally distributed contributions. Furthermore, an intersectional perspective poses the question of whether what is perceived as wellbeing or good care does not neglect specific perspectives. In contrast, an intersectional lens calls for a consideration of the heterogeneity of perceptions of wellbeing and good care, without overlooking — and hence justifying — social inequities and structural injustices. In this way, a critical reflection of the normative goal of intergenerational solidarity from an intersectional perspective can contribute to good care and wellbeing for all — and not to the reproduction of structural injustices under the guise of supposed solidarity.

## Conclusions: Who cares for solidarity in the future?

The COVID-19 pandemic has acted as a magnifying glass for intergenerational debates on healthcare. It can be understood as a stress test for intergenerational relations that offers important lessons for future crises. Our intersectional analysis of the public (re)negotiation of intergenerational relations in the German media highlights the increasing prevalence of gendered ageism in appeals to intergenerational solidarity, particularly in the context of care. It became evident that discursive appeals to solidarity regularly draw on stereotypical assumptions related to different and often entangled social categories. Moreover, solidarity frequently derives its normative force from mechanisms of inclusion and exclusion ([116] p. 308), thus pointing to the “paradox at the heart of solidarity” ([117] p. 103). In our analysis, this applies to all elements of solidarity (*identification*,* group*, *contribution*, and *normative goal*) and underscores the need for further theoretical work on how intergenerational solidarity can be conceptualized in a diversity-sensitive way within different transnational social structures.

Our research demonstrates that approaches from intersectional feminist philosophy can make an important contribution to this endeavour. Applying an intersectional lens to an in-depth case analysis, grounded in a heuristic framework of solidarity, allows a critical extension of the normative basis of solidarity and provides a fundamental tool for understanding the diverse preconditions of solidary action. In turn, this may enable a more normatively justified distribution of burdens associated with intergenerational solidarity in and beyond times of crises. It also allows for a more systematic differentiation of social positions and deepens our understanding of how solidarity intersects, at a structural level, with related moral and political concepts, including responsibility and justice.

By focusing on the intergenerational relations, our research contributes to what has recently been described as an ‘intergenerational turn’ in (bio)ethics (see, e.g., [15, 32, 118, 119]). Thus, a heterogeneous and occasionally rather unreflected reference to originally different strands in the conceptualization of generations can be observed in different domains of bioethics – clinical ethics, public health ethics, and research ethics [32] – on debates on planetary health, global health, or biomedical research [120–122]. Against this backdrop, our analysis highlights the importance of an intersectional, diversity-sensitive perspective on generational approaches in healthcare crises, as well as in medicine and healthcare more broadly. Such a perspective acknowledges heterogeneity within generations while preserving the analytical strength of the concept of generation in bioethics. Further research is needed to advance the conceptual development of ‘generation’ as a social and ethical category.

When interpreting the results of our intersectional analysis, the limitations of the study must be taken into account. First, our study examined two paradigmatic cases from the German media debate. Although these paradigmatic cases were identified within a broader qualitative media debate analysis, further — also quantitative — research is needed to substantiate the hypotheses developed in this study. Furthermore, media debates must not be confused with a direct, quantitative representation of reality, but as a system with powerful actors, mechanisms, inherent incentives and normative positioned (leading) media [84, 88, 123]. It is especially important to consider the character of media contributions in the assessment of our results. At the same time, they provide insights into the hegemony of public discourse and can thus be understood as a particularly fruitful starting point to investigate the effectiveness of social structures in the formation of the “public ethics narrative” [124] — and what is left out in this narrative. Secondly, the focus on the German context warrants consideration. The findings presented here offer a country-specific insight into the media debates in Germany during the COVID-19 pandemic. Whilst these findings cannot be applied to other national contexts in every detail, it is reasonable to assume that the general tendencies in the construction of gendered ageism during the pandemic and our theoretical suggestions also apply to other countries. For example — despite all structural specifities — the Gender Care Gap and a “shecession” or rather a “momcession” could be observed in many other OECD countries as well and, hence, suggest similar mechanisms in appeals to intergenerational solidarity in these countries [125]. Nevertheless, given the global nature of the COVID-19 pandemic and the likelihood of further trans- and international crises, comparative international analyses represent a valuable extension of our research and could help to identify any country-specific nuances of these general trends in comparison to Germany. Finally, while our focus on gender and age as particularly salient social categories already illustrates the importance of an intersectional perspective on intergenerational solidarity, our analysis also points to intersections with other social categories, especially class, sexuality or race, as important determinants for solidary action. Our methodological proceeding and theoretical framework could be used as a toolkit to consider further social categories and to contribute to a more nuanced understanding of how solidarity operates across and within generations by accounting for the interplay of gender, age, and other axes of differentiation.

In light of the multitude of ongoing and emerging crises in the healthcare sector (e.g., nursing crisis) and considering their interconnectedness in times of the *polycrisis* [126, 127], the question of who will care for solidarity between generations in the future and who will bear the burdens of solidarity is crucial. Our analysis shows that it is essential to understand the structural mechanisms that shape a society’s moral fabric so that people across generations, despite their intragenerational diversity, can be in solidarity to confront impending health crises.

## Supplementary Information

Below is the link to the electronic supplementary material.

Supplementary Material 1

## Data Availability

Due to copyright reasons, the full texts of the documents analysed in this study cannot be made publicly available. A list of all documents used in this study can be provided upon reasonable request.

## Footnotes

1 The original German search string was: (Covid ODER Corona ODER Pandemie) UND (solidar* ODER Zusammenhalt* ODER Verbundenheit* ODER Gerechtigkeit* ODER Eigenverantwortung ODER “gesellschaftliche Verantwortung” ODER Konflikt) UND (Generation* ODER Kohorte* ODER Altersgruppe ODER arbeitend* ODER “alt und jung” ODER “die alten” ODER “die jungen” ODER “alte Leute” ODER “alte Menschen” ODER “junge Leute” ODER “junge Menschen” ODER senior* ODER rent* ODER ältere* ODER Pflegeheim ODER schül* ODER student* ODER studier* ODER jüngere* ODER Jugend* ODER Kind*.

2 All quotations from the material have been translated by the authors.
