# Supplementary material for: Gendered forms of intergenerational solidarity in the COVID-19 pandemic: an intersectional perspective on the public renegotiation of a moral resource for future health crises in Germany
Source: Int J Equity Health. 2026 Oct 12;25:244. doi: 10.1186/s12939-026-03014-7 (PMC13663106; doi:10.1186/s12939-026-03014-7)
Supplement: Supplementary file 1 [file 12939_2026_3014_MOESM1_ESM.pdf]

Codebook for the media analysis

Last updated: **20 November 2025**

Methodological remarks:

The ethical concepts brought to bear in the material are not necessarily articulated in moral-philosophical terminology and their conceptual implications cannot be identified without further interpretation. This codebook is intended to provide the basis for an initial overview of the material in which passages for more in-depth, nuanced ethical analyses can be filtered out.

The following applies to all codes: Initially, the texts are to be coded broadly, including sufficient context to facilitate working with the individual text passages in the detailed analysis. As a general rule, codes are applied to passages of at least one sentence. Furthermore, coding is conducted aspect by aspect. This means that a new code is assigned for each additional aspect. The respective coded aspect is noted in a memo. Working hypotheses for initial interpretations are also noted in memos.

Codes	Subcodes	Description
Open Code		Inductive code for topics not already covered by the codebook.
Wave		Instead of coding the specific publishing date of an article, we aim at creating an overview of which topics were discussed during the specific COVID-19 infection waves and how these were being connected to other crises.
	First wave	(03/2020-05/2020)
	Covid summer	(06/2020-09/2020)
	Second wave	(10/2020-02/2021)
	Third wave	(03/2021-07/2021)
	Fourth wave	(08/2021-12/2021)
	Fifth wave	(01/2022-06/2022)

Digitalization		Any text passage referring to processes of digitalization across different sectors should be coded this way. Additionally, aspects of cyberwar and cyberattacks (including those targeting critical infrastructure) fall under this category. Further aspects of social media, such as its impact and role across different generations, should also be included. Furthermore, aspects of hate speech, as well as the concepts of truth and falsity on social media, the internet in general, and the digital world, should be coded this way.
Economy		Any text passage in relation to the economy and finance may or may not be related to crisis topics.
Education		Any text passage that refers to educational topics (school, university etc.).
Migration		Any text passage referring to migration, including emigration, immigration, circular migration, undocumented migration, labour migration and/or educational migration between people of any background to any destination country and from any origin country. This includes policies and policy debates regarding migration, as well as social and cultural topics, including integration and identity. Please also include references to second- and later generation ‘migrants’ if present in the text as well as references to EU freedom of movement.
Sports		Any text passage referring to sports associations, communities, and events, whether professional organizations or more local groups.
Climate Policies		Any text passage that refers to the green transition and adhering climate policy discussion in a broader meaning (not only climate crisis)
Religion		Any text passage that refers to religion in a broader meaning, whether as a cultural system, a moral framework, or a set of beliefs and practices, including religious institutions and communities.
Gender		Any text passage that refers to gender in a broader meaning: gender(ed) conflict(s), stereotypes; claims for diversity; gendered power structures; heteronormativity; queer topics; gendered forms of solidarity; gendered images of age.
Social embeddedness		Any text passage referring to social interactions, community or lack of it, e.g. loneliness, exclusion or marginalization, lockdowns, civil liberties (also in the context of specific

		generations). In this context consequences on the mental health of people because of the lack of social interactions etc. are integrated on this code.
Social Security		Any text passage addressing social security aspects, on the state level (e.g., pensions) as well as institutional support for different groups of individuals (e.g., financial, supply of different resources, subjective perceptions).
Democracy/ other political systems		Any text passage referring to democratic state structures or other political systems (non-democratic): multi-level governance (EU; state level); federal state structure, decision making processes and responsibilities on different political levels; decline/threat to democracy; crisis of democracy; bureaucracy; political responsibilities in general.
Extremism		Any text passage referring extremist behavior or action during the pandemic (corona-protest; conspiracy mythologies; anti-democratic movements; “Querdenken”). This can also refer to other forms of political extremism.
Racism		Any text passage referring to racist decision making or individual or collective racist behavior/action (e.g., Black Lives Matter; measures in the pandemic; distribution of commodities). Broadly related to the code Justice/Injustice.
Temporality		Any text passage referring to historic crises or future crises from the experience/point of view of the current crisis. Furthermore, the reference to specific events in time should be coded.
Solidarity		Any text passage in which a reference to solidarity is made explicitly or implicitly is coded this way. The concept should be understood rather broadly, as outlined by Ellerich-Groppe, Schweda, and Pfaller 2020; Ellerich-Groppe, Pfaller and Schweda 2021. (Keywords that might indicate a reference to solidarity include e.g. community, sense of community, belonging, connection, standing up for each other, connectedness as well as opposite terms such as conflict etc.).
Conflict		Any text passage that refers to conflict as a lack of solidarity and other (inter-) generational conflicts, e.g., in relation to (in)justice.
Responsibility		Any text passage in which the responsibilities of different persons or groups are addressed explicitly or implicitly is coded this way. The concept should be understood rather broadly, as outlined by Ellerich-Groppe, Schweda, and Pfaller 2020; Ellerich-Groppe, Pfaller and Schweda 2021. (Keywords that might indicate a reference to responsibility include: e.g. need to, could, should, may, must, guilt).

	Individual responsibility	Any text passage that refers to the responsibility of an individual person. This responsibility manifests itself in the individual awareness of an obligation to act or behave in a way that has desirable consequences - regardless of whether it benefits the bearer of the responsibility or not. Individual responsibility can show a behaviour or action whose origin can lie in one's own well-being as well as in the interests of others.
	Collective Responsibility	Any text passage that focuses on the responsibility of a collective or group. Characteristics of a collective are a certain degree of structuring, orientation towards a common goal and the ability to act with shared intentions. Collective responsibility is understood as the responsibility of collective actors (as the sum of individual actors) for their actions. Responsible action is categorised as morally culpable or praiseworthy. Examples of collective responsibility can be seen in companies, universities as well as in social organisations. Further, any signs of a button-up approach in relation to responsibility are to be coded this way.
	Institutional/ Governmental responsibility	Any text passage that refers to the responsibility of formal institutions—such as governments, legal systems or public agencies. It encompasses the structured, rule-based obligations that are embedded in the roles, mandates, or legal duties of institutions. Here, a certain top-down approach is recognizable. Along with this, text passages that refer to the government as bearing responsibility, including in matters relating to law abidance and the implementation of decisions. In this way, government may be seen as bearing power of oversight that aims to ensure individuals and collective bodies act in a responsible manner, as well as bearing intrinsic responsibility to ensure the wellbeing of its citizens.
	Systemic/ Unspecified responsibility	Any text passage that does not refer to individuals or collectives as bearers of responsibility (i. e. the subject of responsibility remains unclear), but rather broaden the perspective to include processes that are caused by actions or decisions, but which nevertheless cannot be meaningfully conceptualised as anyone's actions. Rather, the actors are made aware of their situation in a systemic context.
Trust		Any text passage referring to trust in the context of social or political settings, e.g. in the context of broken trust between different generations, in political institutions etc.
Moral concepts		Any text passage that refers explicitly or implicitly to further moral concepts is coded this way (especially if they represent values and norms that could represent the normative goal of solidarity or that may provide the basis for responsibility). (cf. also the relational model in Ellerich-Groppe, Schweda, and Pfaller 2020; Ellerich-Groppe, Pfaller and Schweda 2021; Ellerich-Groppe et al. 2024)

		If necessary and suitable, we may include and code further moral concepts (always upon agreement with the team).
	Freedom/ Autonomy	Any text passage referring to the concept of freedom (positive or negative) and autonomic decision making.
	Care	In contrast to the code ‘well-being’ as the aim of an action/the content of a responsibility, this code refers to the corresponding attitude of actors.
	Justice/ Fairness	Any text passage referring to the concept of justice. This can refer to the conception of justice as fairness as well as distributive justice. Further, references to personal/individual perceptions or experiences as well as institutional/legal procedures are coded this way. As we agreed to code the opposite of moral concepts, reference to injustice or unfairness is also to be coded.
	Wellbeing/ Welfare	In contrast to the code ‘care’ as the attitude of actors, this code refers to the corresponding aim of an action/content of a responsibility.
	Sustainability	Any text passage referring to sustainability in different domains/aspects, e.g. environmental, social, economic, health. This can encompass concrete measures as well as sustainable development (across time).
	Notions of the Good Life	Any text passage referring to conceptions of the good life. This encompasses individual perceptions, experiences, desires, goals or ideas embedded in specific social/cultural settings. These aspects can be situated in time: e.g., across a life span, in a certain moment (e.g. at a specific life stage) or retrospectively. Further, normative ideas of what a good life consists of are to be coded this way.
	(Basic) Rights	Any text passage that refers to (basic) rights as the legal framework of a political system and its individual or social impact (e.g. questions of the social order of human coexistence). This can include aspects of access or deprivation of certain (basic) rights (possible connection to the code justice/fairness) or references to the broader constitutional order. Further, specific legal measures and the exercise of certain rights can be coded this way.

	Dignity	Any text passage referring to a person's dignity, e.g., human dignity. This can relate to (human/basic) rights, such as the right to be treated with dignity across different contexts, and the idea that laws should protect one's dignity within a social setting or society, including institutional settings (e.g., hospitals) and (inter)personal relationships. Dignity can be connected to agency, as in the ability to act with dignity, or vulnerability, such as the need for dignity in situations of illness, dependency, or social exclusion.
Shame/ Guilt/ Blame		Any text passage referring to a moral or psychological framework in which individuals or groups experience, express, and assign responsibility for perceived wrongdoings.
Generation (concept and understanding)		Any text passage that provides indications of the understanding of generation applied is coded this way. This includes the mere mentioning of specific generation names or labels as well as more elaborate and extensive discussions about different notions of generations, such as socio-cultural conceptions, birth-cohorts or familial generations.
	Birth Cohorts/ Age Groups (chronological)	Any text passage that refers to an understanding of generations on the basis of birth cohorts or age groups. In this sense, the stratification of generations occurs according to date and birth. This includes references to *the old* or *the young* as representatives of generations.
	Social/ Historical Generation (socio-cultural)	Any text passage that refers to an understanding of generations as shaped by shared historical, cultural, or social experiences. This includes references to generational identities formed around key events, time periods, or societal changes (e.g. "the post-war generation", "Generation Z", "millennials"). Passages that highlight generational consciousness, generational shifts in norms, or social roles tied to a specific historical moment may also be also be coded this way.
	Familial Generation (genealogical)	Any text passage that refers to an understanding of generation in a familial or kinship sense. This includes identifying members of a familial generation using terms like parent, child, grandparent, grandchild, etc. (also including gendered forms of the preceding, such as mother, daughter, grandfather). Broader references to topics like 'ancestors' and 'family trees' may also be coded this way.
Generational relations		Any text passage that addresses relations between different generations is coded this way. This may include notions about demands, reproaches or calls to action as well as more positive and empathetic remarks on the specific conditions that the other generation has or

		had to experience and endure. We aim at inductively expand this code via sub-codes and to record further thoughts and ideas in memos. Furthermore, this code might also include references to the intragenerational relations within one generation that are renegotiated with reference to intergenerational relations.
Generation-related Narratives		Any text passage that indicates:<br>• distinctive narratives that are used to plausibilize the formation and identity of a generation, e.g. by referring to contemporary or historic events or other narrative sources of identity formation for the respective generations<br>• distinctive narratives about a specific generation as well as attributions that are used as a way to understand or justify this generation's behavior, attitudes or actions and to explain the (moral) relations to other generations.
Images of Ageing		Any text passage that reveals perceptions, representations, and stereotypes about age and ageing that are articulated in the article are coded this way. This comprises both self-ascriptions as well as attributions by others.
Pandemic reference		Any text passage that discusses the COVID-19 pandemic or certain aspects of the pandemic Relevant articles may discuss these pandemic references extensively or rather build a short link to the pandemic (e.g. "as we saw in the pandemic" etc.) With this code, we aim to determine how significant and influential the pandemic was with regards to further crises.
Other crises		Any text passage that addresses further crises beyond the COVID-19 pandemic is inductively coded this way via sub-codes. If necessary, we include memos to specify in more detail in what way the text passage relates to the crisis/code.
	Ukraine war	
	Climate crisis	
	Health Care Crisis	Any text passage referring to the institutional health care crisis, e.g., the NHS
	Nursing Crisis	Any text passage referring to institutional care (e.g. elderly homes) and informal (e.g. caring for family members).

Ellerich-Groppe, Niklas; Pfaller, Larissa; Schweda, Mark (2021): Young for old – old for young? Ethical perspectives on intergenerational solidarity and responsibility in public discourses on COVID-19. *European Journal of Ageing* 18, 159-171. https://doi.org/10.1007/s10433-021-00623-9.

Ellerich-Groppe, Niklas; Schweda, Mark; Pfaller, Larissa (2020): #StayHomeForGrandma – Towards an Analysis of Intergenerational Solidarity and Responsibility in the Coronavirus Pandemic. *Social Sciences & Humanities Open 2*(1), 100085. https://doi.org/10.1016/j.ssaho.2020.100085.

Ellerich-Groppe, Niklas; Steckdaub-Muller, Irmgard; Pfaller, Larissa; Schweda, Mark (2024): Moral Paradigms of Intergenerational Solidarity in the Coronavirus-Pandemic. Analyse & Kritik 46(1), 85-119. https://doi.org/10.1515/auk-2024-2010.
